# Hand-suturing–assisted traction technique and closure in colon endoscopic submucosal dissection

**DOI:** 10.1016/j.vgie.2024.10.010

**Published:** 2024-10-21

**Authors:** Fatih Aslan, Serhat Ozer, Volkan Demirdogen

**Affiliations:** 1 Department of Gastroenterology and Advanced Endoscopy, Koc University Hospital, Istanbul, Turkey; 2 Department of Anesthesiology and Reanimation, Amerikan Hospital, Istanbul, Turkey

## Introduction

Colorectal endoscopic submucosal dissection (ESD) is one of the effective and minimally invasive methods in en bloc and curative resection of premalignant and early-stage malignant lesions more than 2 cm in size.^1^ However, certain variables, including lesion size, being in proximal colon or flexures, and fibrosis, make the procedure difficult to complete.^2^ Other factors complicating the quality and success rate of the procedure include poor maneuverability in proximal segments, gravitational issues, and early bleeding or perforation.^3^ In addition, not closing the ESD site, particularly where the muscularis propria are quite thin, may lead to postpolypectomy syndrome and late perforation.^4^ We, herein, present a cecal ESD case in which previous hazards are overcome with a simple and cost-effective approach.

## Case

**Figure 1:**
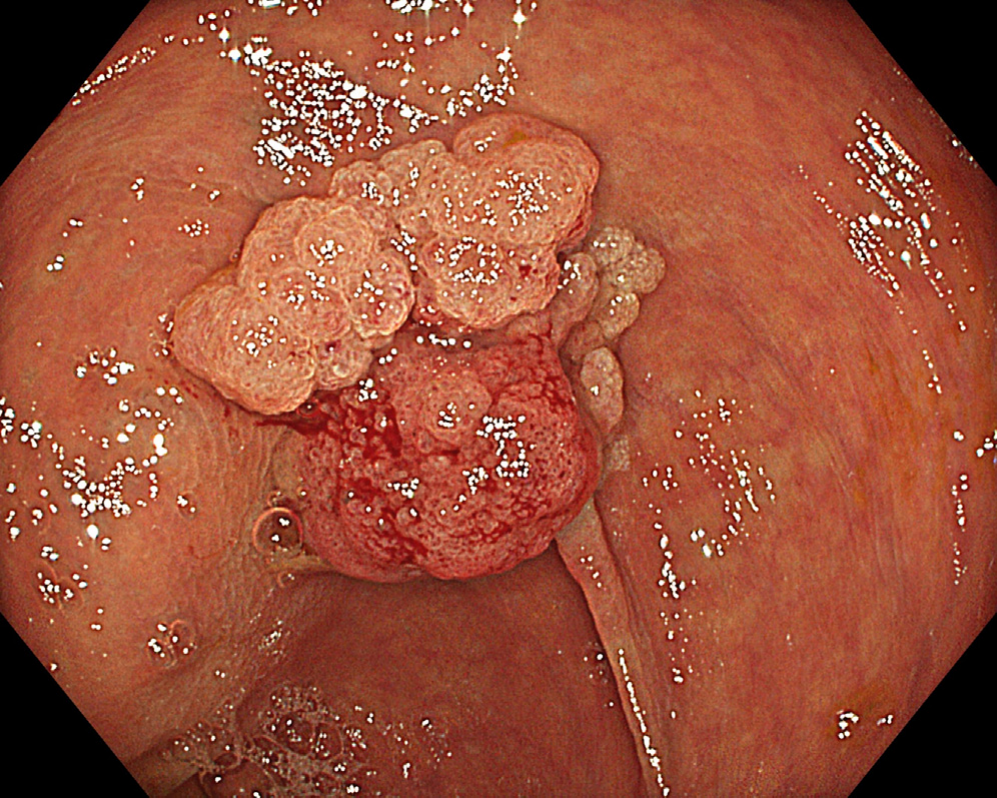
The endoscopic appearance of an LST-GM type lesion using the TXI mode. *LST-GM*, laterally spreading tumor, granular-mixed; *TXI*, Texture and color enhancement imaging.

**Figure 2:**
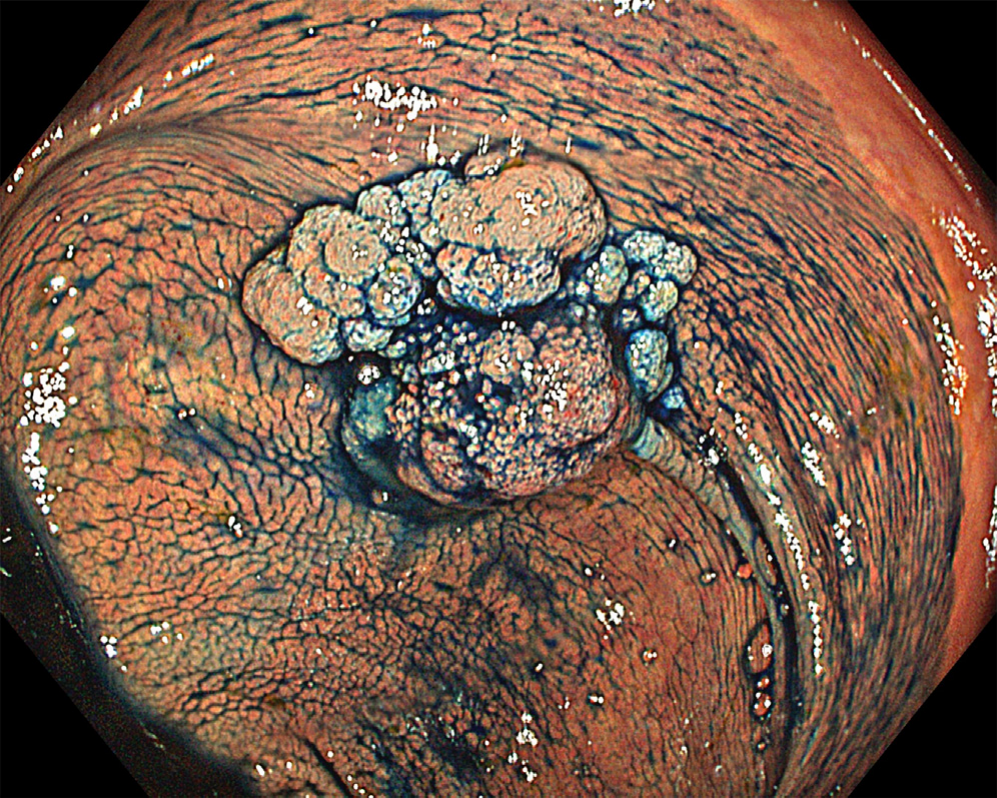
The endoscopic appearance of an LST-GM type lesion after indigocarmine staining. *LST-GM*, laterally spreading tumor, granular-mixed.

**Figure 3:**
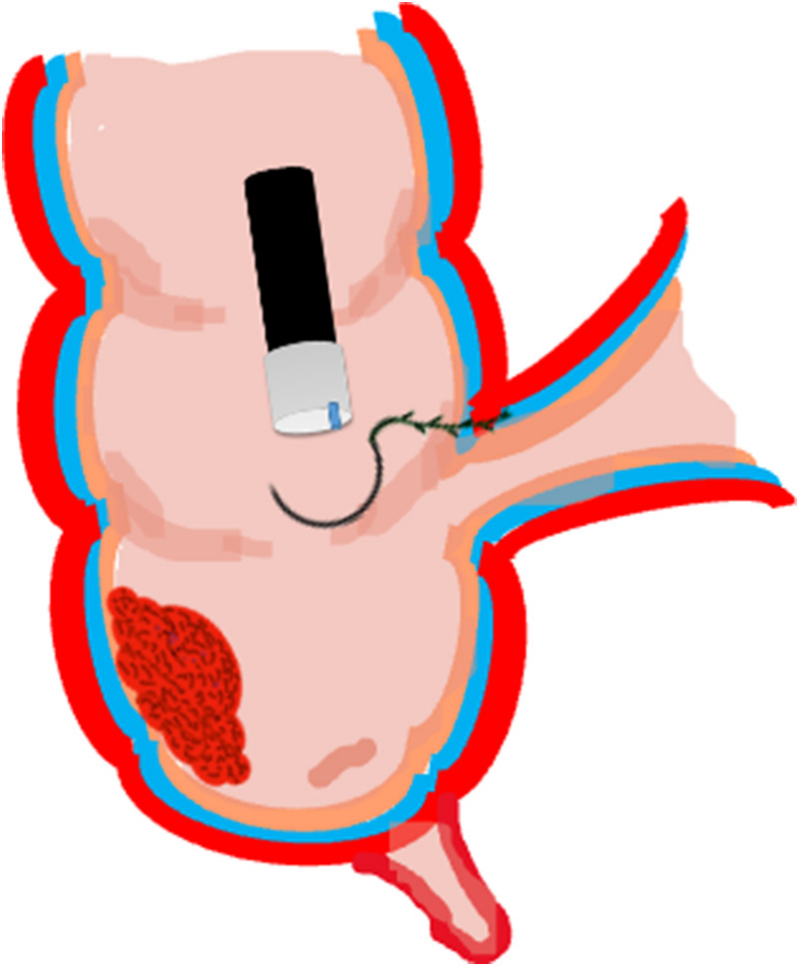
Transporting the barbed suture to the ascending colon.

**Figure 4:**
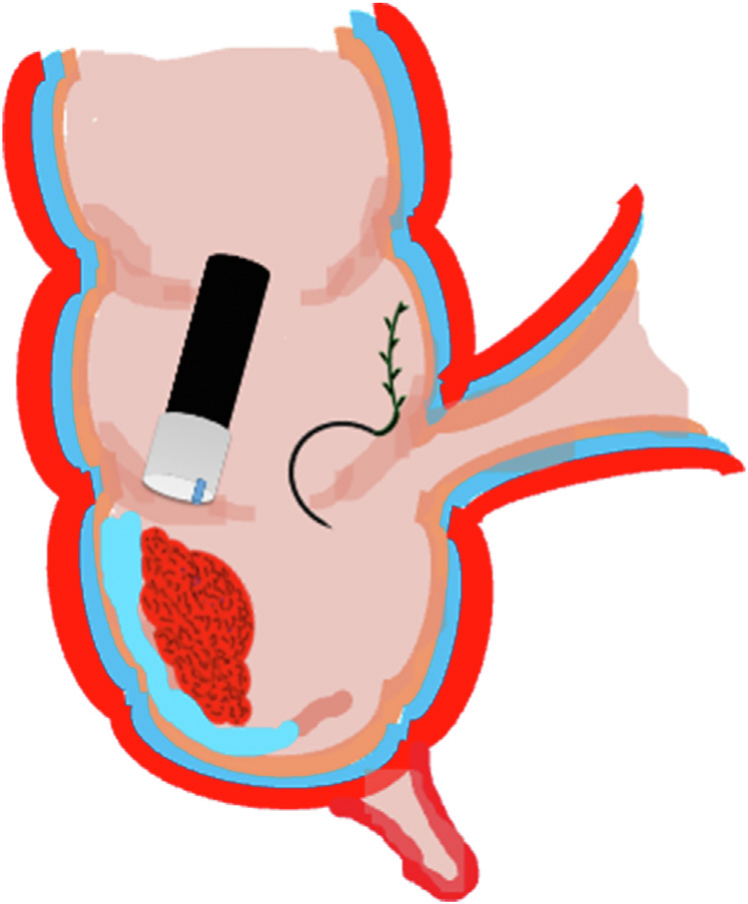
Schematic view of the submucosal injection and circumferential mucosal incision.

**Figure 5:**
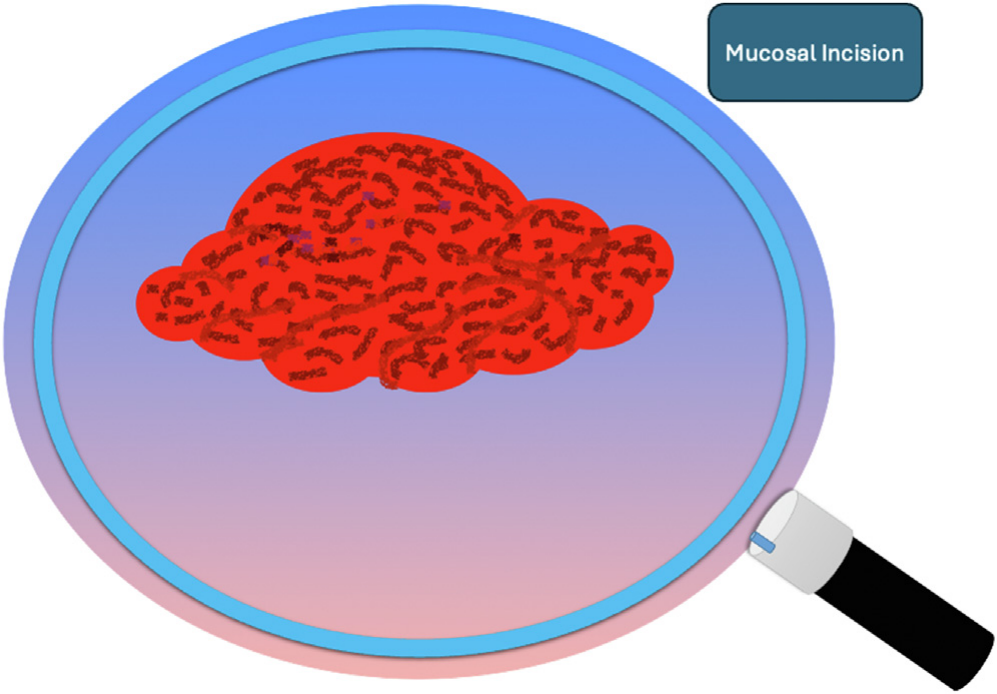
Schematic view of the circumferential mucosal incision.

**Figure 6:**
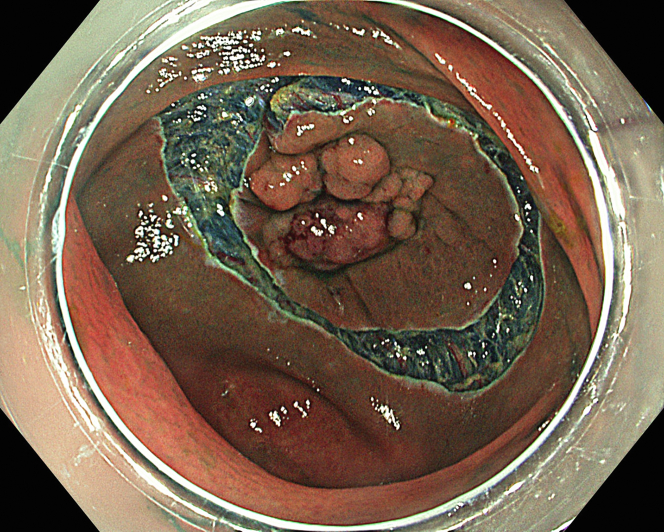
The endoscopic view of circumferential mucosal incision.

**Figure 7:**
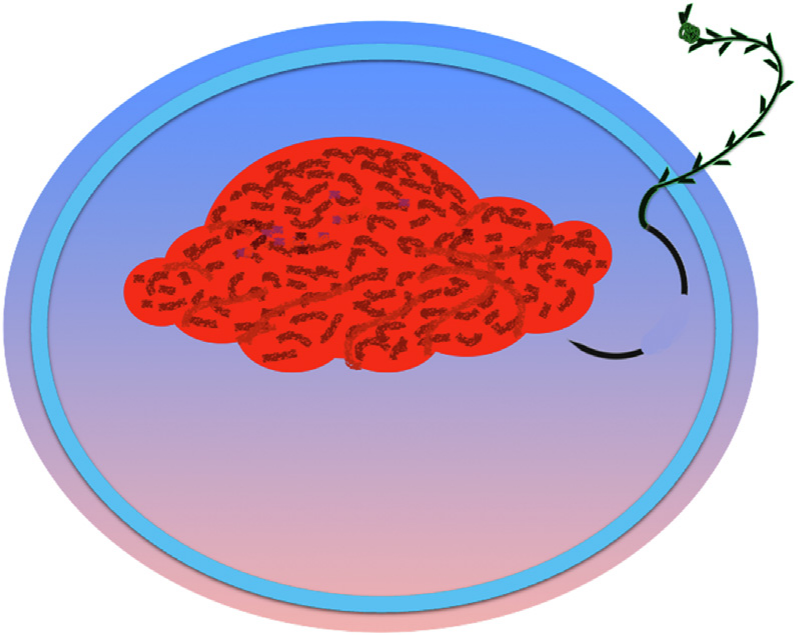
Passing the barbed suture through the mucosa and submucosa.

**Figure 8:**
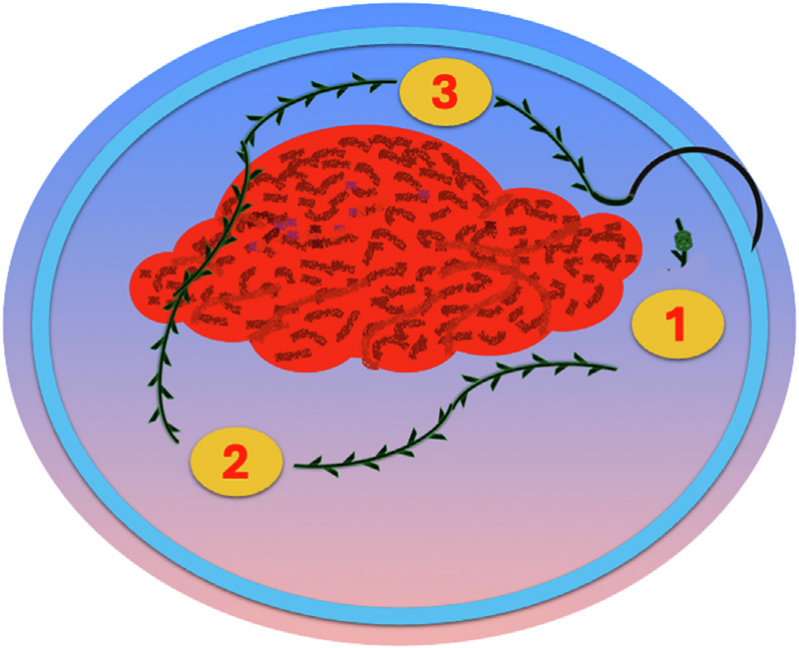
Passing the barbed suture through 3 different areas of the lesion.

**Figure 9:**
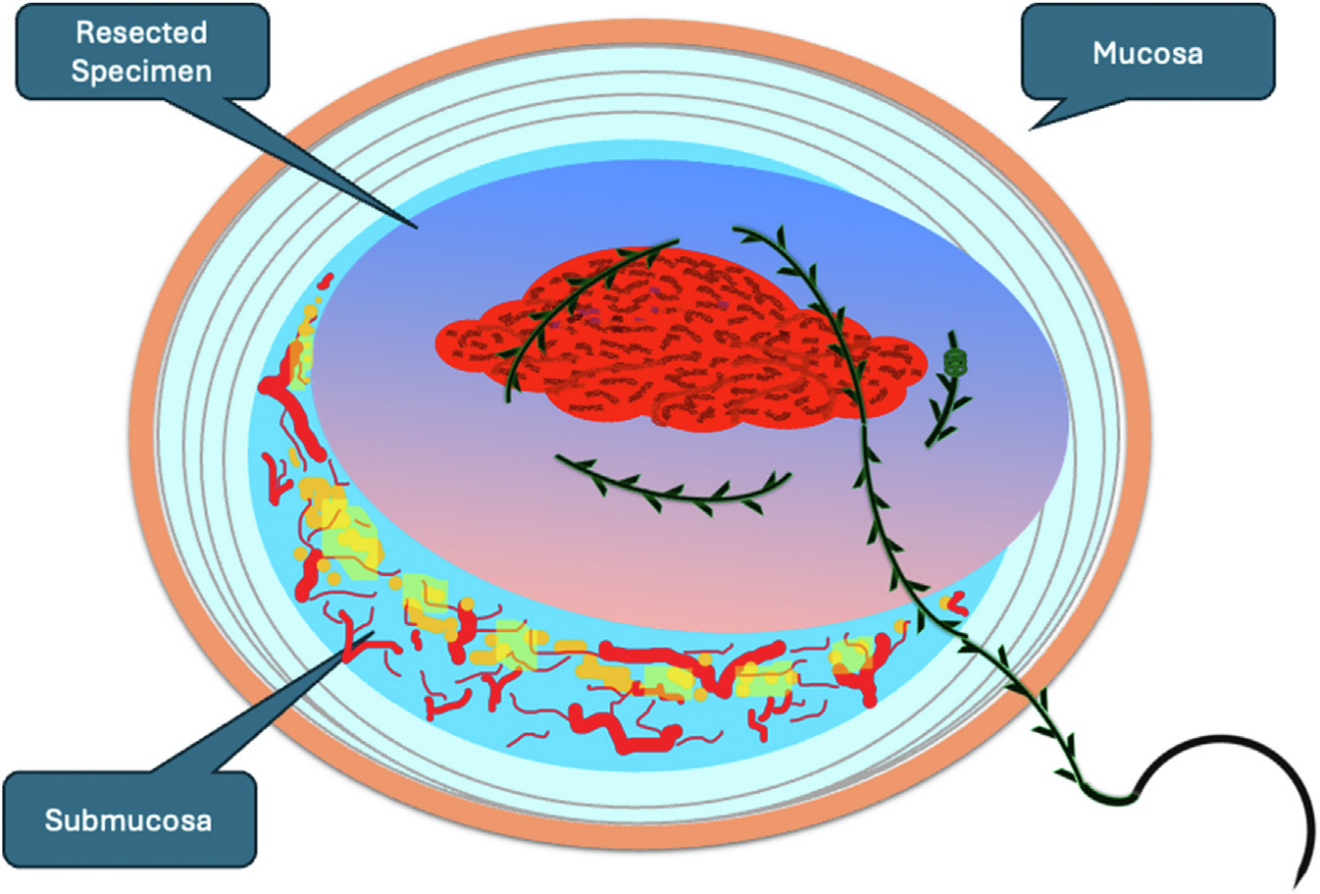
Tightening the suture and passing it through the proximal colon fold to apply traction to the lesion.

**Figure 10:**
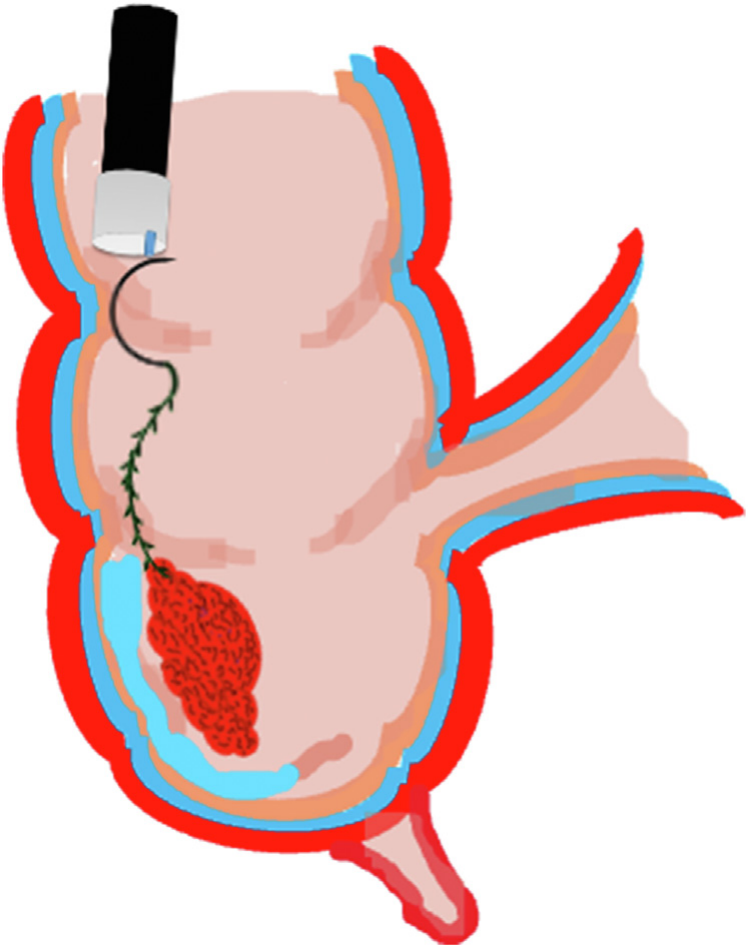
Applying traction to the lesion using the barbed suture.

**Figure 11:**
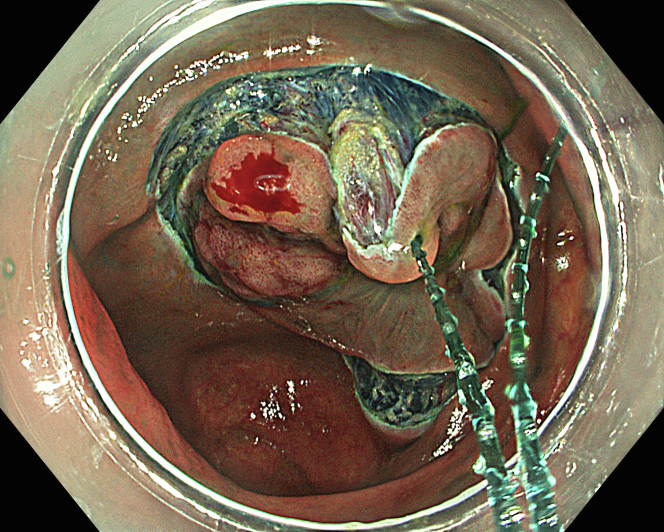
The appearance of the lesion during the ESD procedure while traction is applied. *ESD*, Endoscopic submucosal dissection.

**Figure 12:**
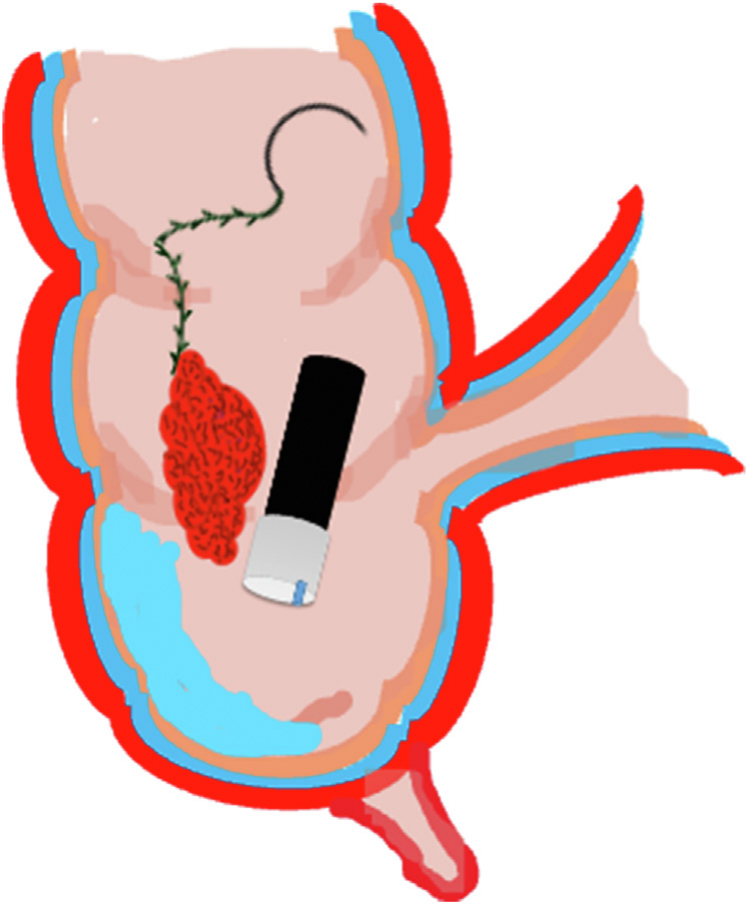
Continuing dissection after traction is applied.

**Figure 13:**
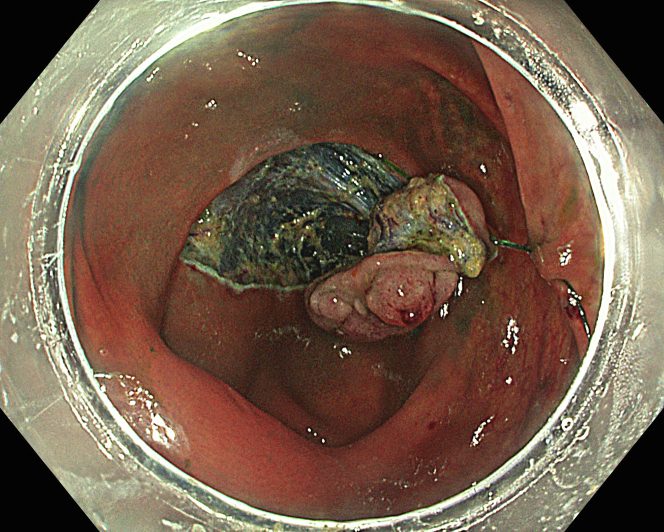
The appearance of the lesion during the ESD procedure while traction is applied at different times. *ESD*, Endoscopic submucosal dissection.

**Figure 14:**
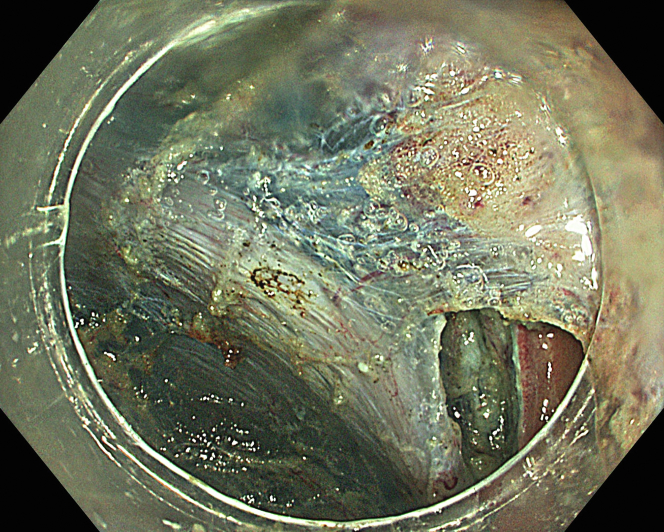
The appearance of the lesion during the ESD procedure while traction is applied at different times. *ESD*, Endoscopic submucosal dissection.

**Figure 15:**
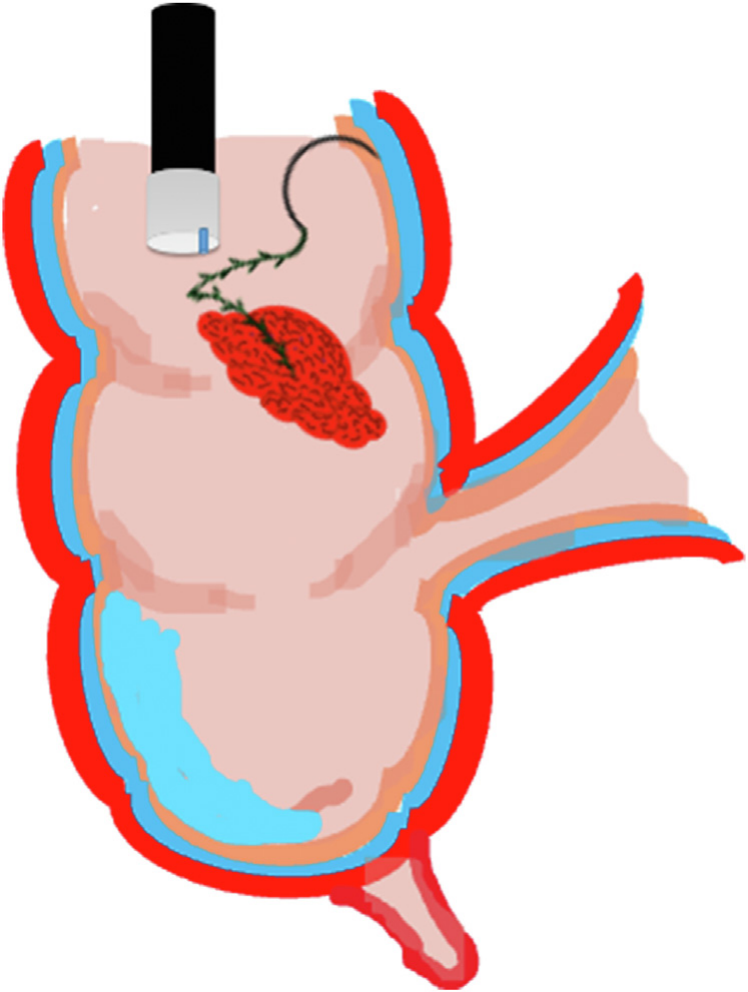
Removal of the resected lesion and remaining barbed suture.

**Figure 16:**
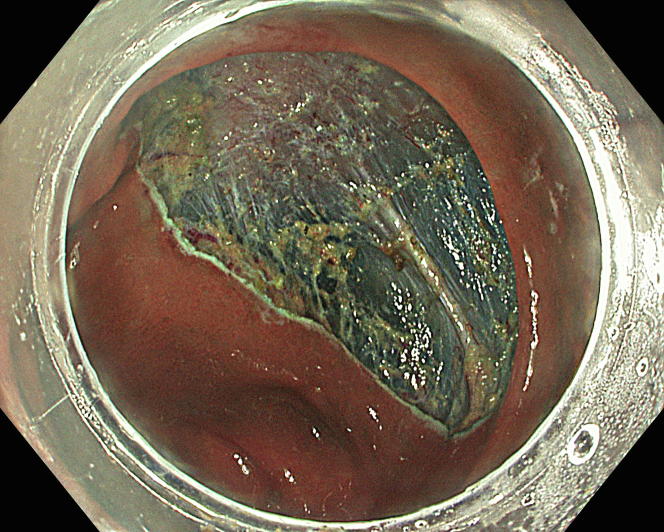
The appearance of the resection site after the procedure.

**Figure 17:**
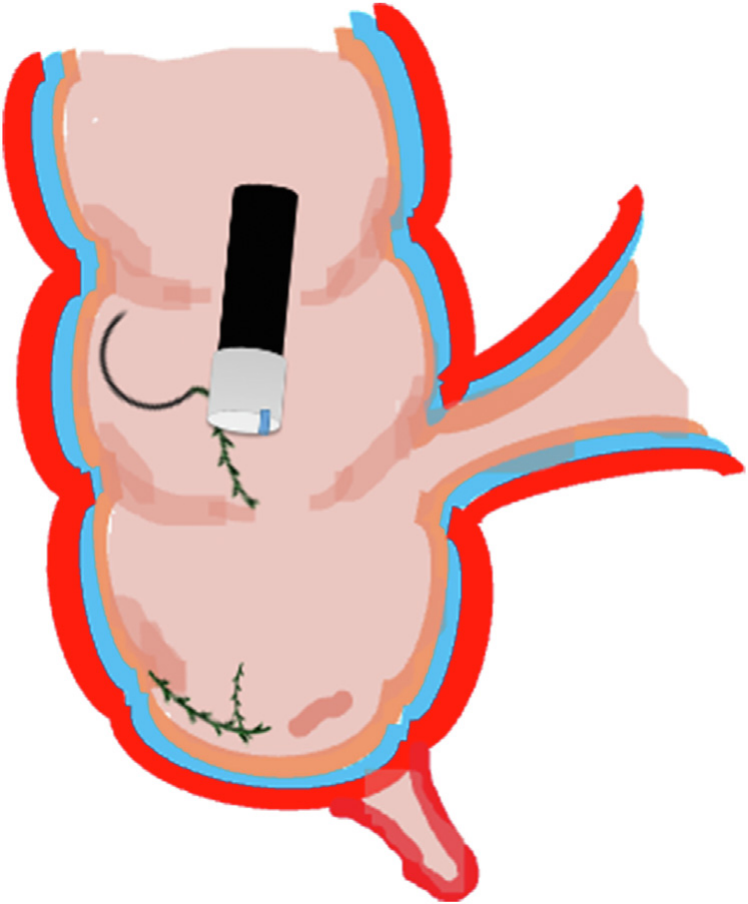
Endoscopic closure of the resection site with the remaining barbed suture.

**Figure 18:**
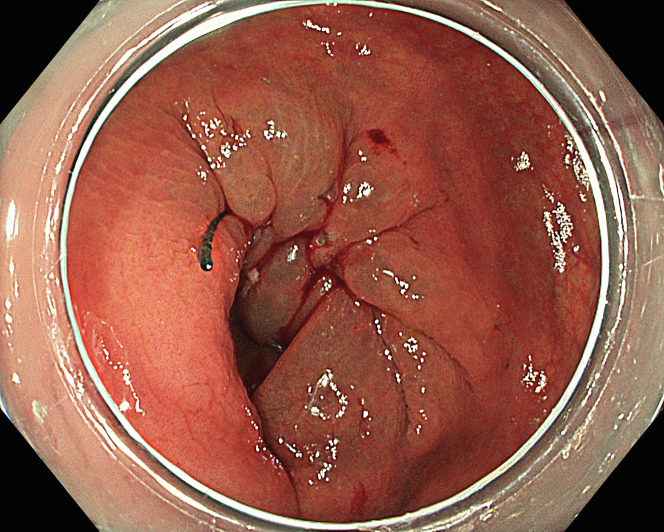
The appearance after endoscopic closure with the barbed suture.

**Figure 19:**
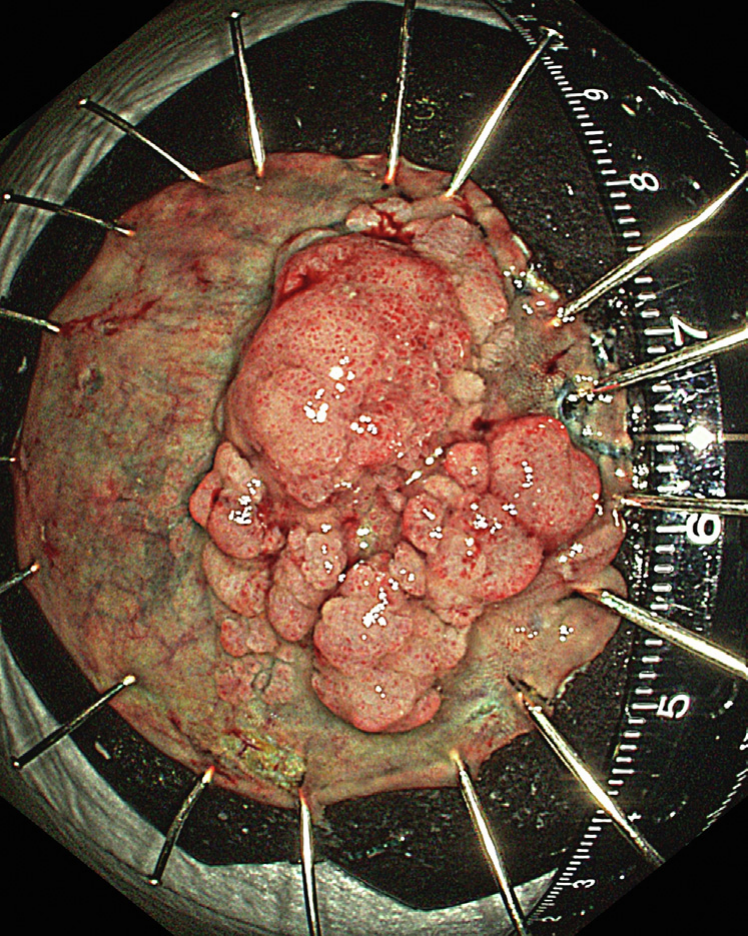
The endoscopic appearance of the resected material using TXI mode. *TXI*, Texture and color enhancement imaging.

A 47-year-old male patient presented with a positive fecal occult blood test. On colonoscopy, a 3-cm mixed-type laterally spreading tumor in the cecum with an irregular vascular and surface pattern with respect to white-light imaging, texture and color enhancement imaging, narrow-band imaging, and indigo carmine–assisted chromoendoscopy was noted (Figs. 1 and 2). Given the positional issues, a hand-suturing traction technique was chosen for fast and safe dissection.^5^ An absorbable barbed suture (V-Loc 180, 2-0, CV-23; Medtronic Ltd, Dublin, Ireland), with a ring-shaped needle on the tip, which is routinely in use in surgical procedures, was planned for use in traction. Given the length of the suture, it was cut and shortened. Three knots were tied to better prevent detachment. Then, the barbed suture was transported to the area of the lesion within a translucent hood with the help of Needle Holder (SutuArt FG-260U; Olympus, Tokyo, Japan), which already had been passed through the working channel of the colonoscope (Video 1, available online at www.videogie.org). The barbed suture with a needle was fixed to the healthy mucosa close to the ileocecal valve to prevent unwanted damage (Fig. 3). Then the procedure started with lifting using a sclerotherapy needle (Needle Master; Olympus), Voluven (Fresenius Kabi AG, Bad Homburg, Germany), and indigo carmine. Circumferential mucosal incision was completed with Dual Knife (Olympus) under pulse-cut slow mode (Watt 40, Effect 2) (ESG300; Olympus) (Figure 4, Figure 5, Figure 6). Three consecutive insertions using a needle holder and barbed suture were applied to the already circumferentially incised and lifted target tissue that included the mucosa and submucosa but not the muscularis propria (Figure 7, Figure 8, Figure 9, Figure 10, Figure 11). Then, the barbed suture was pulled back with the needle holder, resulting in shrinkage and traction of the lesion, which led to a safe and fast dissection with a dual knife under the same electrocautery settings (Figure 12, Figure 13, Figure 14, Figure 15). Next, the suture was cut with endoscopic scissors close to the traction point (Loop Cutter; Olympus). Resection material with the barbed suture embedded was taken out en bloc. Traction and dissection time were 4.5 and 16 minutes, respectively. No adverse event was seen. After the resection, the ESD area was closed with the same suture having 3 previous knots at the distal tip (Figure 16, Figure 17, Figure 18). Given the antislip feature of the suture, no extra knot was tied after the last insertion and the remainder was cut and removed. The patient was discharged after 4 hours. R0 resection was achieved with negative lateral and vertical borders with an adenocarcinoma 47 × 45 mm in size (Fig. 19) (Video 1, available online at www.videogie.org).

## Discussion

Colorectal ESD, an advanced endoscopic procedure, may sometimes be challenging given anatomical inconveniences or different lesion characteristics with a quite long learning curve.^6^ Besides, it has a higher adverse event rate than polypectomy or EMR. High adverse event rates may sometimes discourage endoscopists from performing this procedure and make them switch to conventional methods, which may result in low en bloc resection and high recurrence rates.^7^ Various ESD techniques and equipment were developed to overcome these negative points and increase en bloc resection rates.^8^, ^9^, ^10^ However, these novel techniques and equipment may incur high costs and are not always easy to access.

In our case, the barbed suture, almost always available in many centers, was carried to the cecum with a needle holder to easily pull the target area that had already been circumferentially incised, which resulted in apparent submucosal tissue to be dissected. Thus, dissection was completed shortly even if the knife was perpendicular to the muscularis propria most of the time. In addition, no hemostatic clip, reported to be used in most traction methods, was used.^11^ Also, detachment of the suture during traction may easily be fixed with a new insertion point using the same equipment, with no additional cost.

Perforation, bleeding, or electrocautery syndrome is among the late adverse events after ESD, which extend hospital stay.^12^ Any instant damage risk to the muscularis propria, at its thinnest point, was prevented via traction in our case, which indirectly lowered the risk of delayed events with diminished thermal injury. In addition, closure of the resection area with the same equipment enabled further safety and cost-effectiveness.^13^

Consequently, the hand-suturing traction method, with easily accessible equipment, provides safety during and after the procedure, and encourages endoscopists to perform difficult ESDs. We are of the opinion that with time this technique and equipment will be used not only in resections but also in various advanced procedures.

## Disclosure

The authors disclosed no financial relationships.

## Supplementary data

Video 1 Hand-suturing–assisted traction technique and closure in colon endoscopic submucosal dissection.
